# Nodal-Dependent Mesendoderm Specification Requires the Combinatorial Activities of FoxH1 and Eomesodermin

**DOI:** 10.1371/journal.pgen.1002072

**Published:** 2011-05-26

**Authors:** Christopher E. Slagle, Tsutomu Aoki, Rebecca D. Burdine

**Affiliations:** Department of Molecular Biology, Princeton University, Princeton, New Jersey, United States of America; University of Pennsylvania, United States of America

## Abstract

Vertebrate mesendoderm specification requires the Nodal signaling pathway and its transcriptional effector FoxH1. However, loss of FoxH1 in several species does not reliably cause the full range of loss-of-Nodal phenotypes, indicating that Nodal signals through additional transcription factors during early development. We investigated the FoxH1-dependent and -independent roles of Nodal signaling during mesendoderm patterning using a novel recessive zebrafish *FoxH1* mutation called *midway*, which produces a C-terminally truncated FoxH1 protein lacking the Smad-interaction domain but retaining DNA–binding capability. Using a combination of gel shift assays, Nodal overexpression experiments, and genetic epistasis analyses, we demonstrate that *midway* more accurately represents a complete loss of FoxH1-dependent Nodal signaling than the existing zebrafish *FoxH1* mutant *schmalspur*. Maternal-zygotic *midway* mutants lack notochords, in agreement with FoxH1 loss in other organisms, but retain near wild-type expression of markers of endoderm and various nonaxial mesoderm fates, including paraxial and intermediate mesoderm and blood precursors. We found that the activity of the T-box transcription factor Eomesodermin accounts for specification of these tissues in *midway* embryos. Inhibition of Eomesodermin in *midway* mutants severely reduces the specification of these tissues and effectively phenocopies the defects seen upon complete loss of Nodal signaling. Our results indicate that the specific combinations of transcription factors available for signal transduction play critical and separable roles in determining Nodal pathway output during mesendoderm patterning. Our findings also offer novel insights into the co-evolution of the Nodal signaling pathway, the notochord specification program, and the chordate branch of the deuterostome family of animals.

## Author Summary

Multiple signaling pathways function combinatorially to form and pattern the primary tissue layers of almost all organisms, by interacting with each other and by utilizing different pathway components to perform specific roles. Here we investigated the combinatorial aspects of the Nodal signaling pathway, which is essential for proper induction of mesoderm and endoderm in vertebrates. We identified a new mutation in the zebrafish *FoxH1* gene, which encodes a Nodal pathway transcription factor, a protein that responds to Nodal signals to carry out the pathway's cellular functions by regulating target gene expression. Using this mutation, we determined that FoxH1 acts in a combinatorial fashion with two other transcription factors, called Mixer and Eomesodermin, to carry out all roles of the Nodal pathway during early development. Through genetic manipulation, we were able to identify the discrete functions regulated by different combinations of these three transcription factors. Our results indicate that the availability of specific Nodal-responsive transcription factors dictates the functions of the Nodal pathway in specific areas of the developing embryo. Our work also provides evidence that the FoxH1 family of transcription factors evolved concomitantly, and perhaps causally, with the chordate branch of animals, to which all vertebrates including humans belong.

## Introduction

The Nodal signaling pathway performs several key steps during vertebrate development. Nodal signals are required for the initial specification and animal-vegetal patterning of mesoderm and endoderm. Nodal is also crucial for induction of the dorsal organizer, a specialized tissue that secretes a host of signals to pattern mesodermal fates along the dorsal-ventral axis and to induce the neuroectoderm [1]. During gastrulation, Nodal signals are maintained in the notochord and prechordal plate, the dorso-axial derivatives of the organizer. These structures are crucial for patterning the neural tube and brain, events which also involve Nodal signals. Finally, asymmetric Nodal activation during somitogenesis governs the laterality of organs such as the gut and heart, and asymmetric lobe development of mammalian lungs. The dependence of the embryo on proper Nodal signaling is evidenced clearly in zebrafish by double mutants for the Nodal homologs *cyclops* and *squint* and by maternal-zygotic (MZ) *one-eyed pinhead* (*oep*) mutants. These mutants, which entirely lack either the two early zebrafish Nodals or the essential extracellular EGF-CFC coreceptor, respectively, exhibit no Nodal signaling and consequently a near-complete loss of mesoderm, an absence of endoderm, and a severe disruption in neural patterning [2], [3].

Many components of the Nodal pathway have been identified and characterized in addition to the EGF-CFC coreceptor [4], [5]. The Nodal family of ligands belongs to the activin-like subgroup of the TGF-ß superfamily and shares many signaling components with other activin-like pathways. These common components include the type I and II activin receptors, Alk4 and ActRIIA/B respectively, the receptor-activated Smads, Smad2/3, and the effector Smad, Smad4. The *Xenopus FoxH1* gene encodes the first transcription factor found to bind to activated Smads in response to activin-like signaling [6]. A Forkhead-family transcription factor conserved across vertebrate species, FoxH1 activates several Nodal targets, including *nodal* homologues themselves, the *lefty* Nodal inhibitors, and several mesendoderm-specific transcription factors, including *goosecoid* (*gsc*), *no tail* (*ntl*)/*brachyury*, the zebrafish *floating head* (*flh*) gene, and certain members of the Mix/Bix family of paired-like homeodomain factors [7]. Loss of FoxH1 function, through a targeted knockout in mouse or morpholino knockdown in *Xenopus*, causes a significant reduction in head structures and a complete loss of axial mesoderm [8]–[11]. These defects are similar to, but less severe than, those caused by a complete loss of Nodal signaling, suggesting that other transcription factors can also activate Nodal targets.

Two alleles of the zebrafish mutant *schmalspur* (*sur*) were independently isolated from two ENU mutagenesis screens [12]–[14]. The *sur* alleles were mapped to the *FoxH1* locus and found to encode single-nucleotide substitutions ten bases apart from each other, leading to an Arg→His (*FoxH1^m768^*) or a Lys→Asn (*FoxH1^ty68b^*) at the beginning of the Forkhead DNA-binding domain [15], [16]. Due to the mutations' positions in the FoxH1 polypeptide and the failure of both mutant proteins to activate a luciferase reporter linked to FoxH1 binding sites [17], the *sur* alleles have been assumed to represent null mutations of *FoxH1*. However, embryos possessing both maternal and zygotic *sur* alleles display only mild versions of FoxH1 loss-of-function phenotypes observed in other organisms, including variable deficiencies in axial mesoderm and floor plate, as well as variable degrees of synopthalmia/cyclopia [15], [16]. The relatively mild defects of MZ*sur* compared to *FoxH1* loss in *Xenopus* and mouse led to the speculation that another Smad-interacting transcription factor, such as the zebrafish *Mixer* homologue *bonnie and clyde* (*bon*), can partially compensate for the *sur* mutation [18].

In this study we describe a novel mutation in zebrafish *FoxH1*, named *midway* (*mid*). This mutation causes highly penetrant defects in axial mesoderm specification that are significantly stronger than those of *sur*. Early molecular markers for, and later morphogenesis of, axial mesoderm are severely reduced or absent in MZ*mid* embryos. These phenotypes more closely resemble loss of FoxH1 function in other organisms, suggesting that FoxH1 has a conserved role in axial development among all vertebrate species. Furthermore, investigation into the differences between the MZ*mid* phenotypes and those caused by a complete loss of Nodal signaling provides new insights into the functions of the Nodal pathway during mesendoderm induction and patterning. FoxH1 function is required for notochord formation but is dispensable for most nonaxial mesoderm fates, which appear to rely on Eomesodermin (Eomes) activity for their earliest specification. Early endoderm induction also does not strictly require FoxH1, instead depending on Eomes and Bon. All three transcription factors contribute to gene expression in the organizer/prechordal plate and subsequent anterior neural development. Our results lead to a model in which the roles of Nodal during early development are partially distinct and separable according to the transcription factor or factors used by the responding cells.

## Results

### The *midway* (*mid*) allele encodes a novel mutation of *FoxH1*

The *FoxH1^Pr1^* allele (which we refer to as *midway*) was isolated as a spontaneously occurring recessive mutation exhibiting a ventral body curvature at 24 hours post-fertilization (hpf; data not shown). Initial morphological analysis revealed that *mid* homozygotes failed to undergo cardiac jogging (data not shown), prompting the name *midway* and suggesting that the *mid* mutation perturbs the process of left-right patterning. RNA *in situ* hybridization analysis for *southpaw*, a *Nodal* homologue that is the earliest left-right asymmetrically expressed gene known in zebrafish [19], revealed a complete absence of expression in the lateral plate mesoderm (data not shown). These phenotypes closely resembled those caused by the *sur* alleles of the *FoxH1* gene [20]–[22].

Bulked segregant analysis followed by genetic mapping by recombination frequency [23] supported the identity of *mid* as an allele of *FoxH1*. *mid* mapped to a roughly 10-cM interval on chromosome 12, defined by SSLP markers z27025 and z11549, that included the *FoxH1* locus. The phenotypes and mapping data prompted a complementation analysis between *mid* and *sur* (*FoxH1^m768^*, the strain we used for all subsequent experiments involving *sur*) heterozygotes. These matings consistently produced clutches in which roughly 25% of the embryos exhibited ventral body curvature similar to homozygotes of either allele, indicating that *mid* and *sur* are in the same complementation group (data not shown). As final confirmation of the identity of the *mid* locus, *FoxH1* mRNA transcribed from a pCS2 expression vector containing the full-length *FoxH1* cDNA was microinjected into *mid* heterozygote incross progeny at the one-cell stage. Injection of 10 pg *FoxH1* mRNA reduced the occurrence of ventral body curvature from 23% in uninjected clutches (n = 162) to 5% (n = 313). The phenotypes, *sur* complementation failure, mapping results, and rescue injections together indicate that the *mid* mutation lies within the *FoxH1* gene.

**Figure 1:**
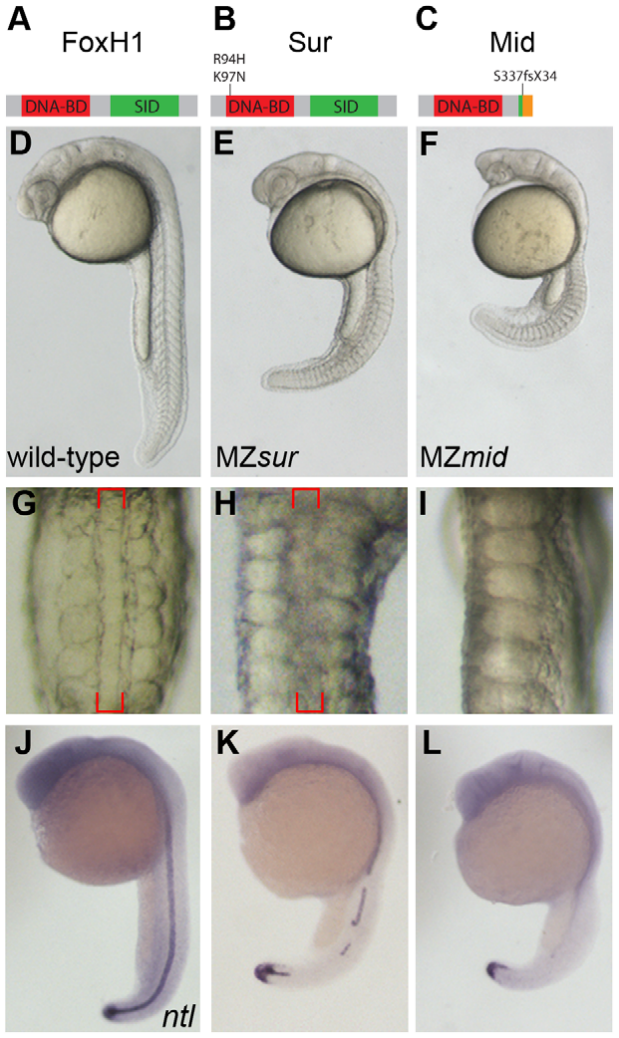
MZ*mid* mutants lack notochords. (A–C) Protein diagrams of *FoxH1* alleles. (D–F) 24 hour post-fertilization (hpf) images of live wild-type and maternal-zygotic *FoxH1* mutant embryos. (G–I) Dorsal zooms (2.5× relative magnification) of embryos in D–F at the level of the yolk extension. Red brackets mark the notochord; MZ*mid* mutants lack this structure and exhibit midline-fused somites. (J–L) RNA *in situ* hybridization for *ntl* expression in 24 hpf wild-type and maternal-zygotic *FoxH1* mutants.

To identify the molecular lesion in the *mid* allele, we sequenced the genomic *FoxH1* locus in *mid* mutants. Importantly, the missense mutations of the two *sur* alleles were not present in the *mid* locus, distinguishing the *mid* and *sur* lesions at the molecular level. We discovered a two-nucleotide insertion at the beginning of the Smad-interaction domain (SID) which causes a frameshift at residue 337 of the 472-amino acid polypeptide. This frameshift causes a truncation that would eliminate all but the most N-terminal three amino acids of the SID, presumably prohibiting the resulting truncated FoxH1 protein from mediating any Smad-transduced transcriptional responses (Figure 1A–1C).

### Axial mesoderm induction is differentially disrupted in *FoxH1* mutants

*FoxH1* is supplied to oocytes as a maternal mRNA, and MZ*sur* embryos display stronger phenotypes than their zygotic counterparts (Z*sur*). These defects include variable deficiencies in axial mesoderm-derived tissues, particularly the notochord and prechordal plate, the latter leading to variable synopthalmia [15], [16]. We therefore wanted to compare the severity of MZ*mid* phenotypes to the defects observed in MZ*sur* mutants. To do so, we rescued *mid* homozygous embryos by *FoxH1* mRNA injection and genotyped them as adults to verify the identities of the homozygous mutants. The genotyped mutants were then mated with each other to produce clutches consisting exclusively of MZ*mid* embryos.

In contrast to the variable defects of MZ*sur* mutants, MZ*mid* embryos consistently display a highly penetrant absence of notochord and full cyclopia, hallmarks of Nodal signaling deficits (172/172; Figure 1D–1I). The loss of a morphological notochord is corroborated by a complete absence of the notochord marker *ntl* at 24 hpf in the midlines of MZ*mid* mutants (71/72), whereas midline *ntl* expression was observed in a discontinuous pattern in a majority of MZ*sur* embryos (35/51) (Figure 1J–1L), with the remainder exhibiting strong continuous midline *ntl* expression. Similar results were seen for other notochord markers, such as *flh*, *sonic hedgehog* (*shh*), and *collagen2a* (*col2a*) (data not shown).

**Figure 2:**
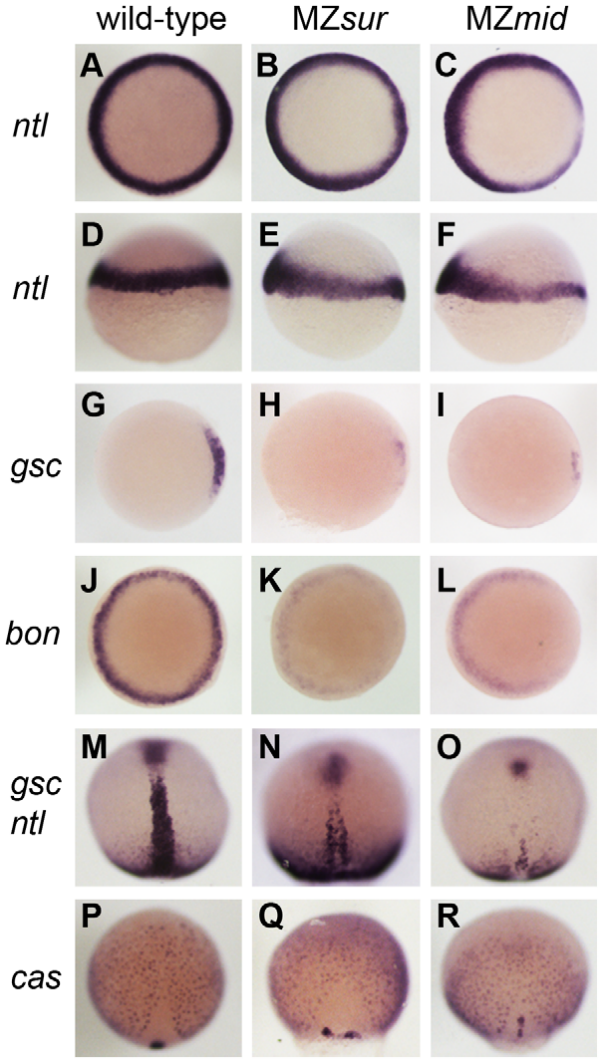
Nodal-dependent tissue specification is differentially disrupted in *FoxH1* mutants. (A–L) Mesendoderm marker expression in wild-type and maternal-zygotic *FoxH1* mutants at 40–50% epiboly. Dorsal is to the right; A–C, G–L are animal views, D–F are lateral views. Note the dorsal reduction of *ntl* expression in MZ*sur* (E) and MZ*mid* (F). (M–R) Axial mesoderm and endoderm marker expression at 90% epiboly, viewed dorsally with anterior up.

To determine how early these defects were first apparent in MZ*mid* mutants, we compared dorsal mesoderm marker expression in MZ*mid* and MZ*sur* mutants at 50% epiboly and 90% epiboly (Figure 2). At 50% epiboly, all MZ*sur* and MZ*mid* embryos display a significant thinning of the *ntl*-expressing dorsal margin (MZ*sur* n = 81; MZ*mid* n = 87) and a significant reduction of *gsc* expression (MZ*sur* n = 60; MZ*mid* n = 68) (Figure 2A–2I). However, at 90% epiboly, MZ*sur* embryos show significant, though abnormal, midline expression of *ntl* (40/40) and *gsc* (34/34), whereas MZ*mid* embryos almost completely lack midline *ntl* expression (63/63) and have less *gsc* expression (44/44) than MZ*sur* (Figure 2M–2O). Other gastrulation-stage notochord markers, including *axial*, *flh*, *shh*, and *lefty1*, are also reduced in MZ*sur* but almost completely absent in the midlines of MZ*mid* (data not shown). Note that the endodermal marker *cas* is retained in both MZ*sur* and MZ*mid* (see below). These early phenotypes indicate a defect in the initial specification of the chordamesoderm in *FoxH1* mutants. Therefore, our results not only strongly suggest that *mid* represents a stronger loss of FoxH1 function than *sur*, they also reveal an absolute requirement for FoxH1 in zebrafish axial specification.

### *FoxH1* mutants respond differently to Activin-like signaling

**Figure 3:**
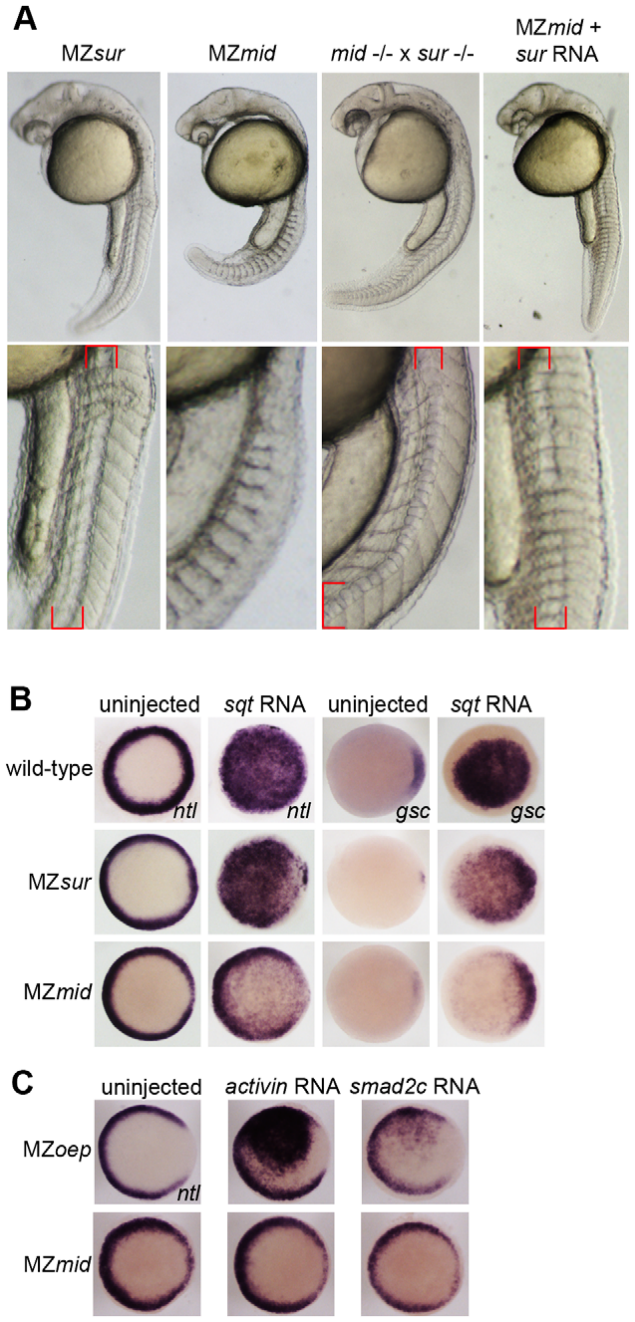
*sur* retains more Nodal transduction capability than *mid*. (A) Genetic interactions between the *sur* and *mid* alleles, demonstrating the ability of *sur* to partially rescue the loss of notochord caused by *mid*. Lower panels are enlargements showing the structures of the notochords at 24 hpf; red brackets indicate notochord domains. (B) Nodal overexpression in maternal-zygotic *FoxH1* mutants. 50 pg *sqt* RNA was injected into wild-type, MZ*sur*, and MZ*mid* embryos at the one-cell stage, and embryos were assayed for Nodal target gene expression at 30–40% epiboly. Note the greater ability of MZ*sur* embryos to respond to ectopic *sqt* compared to the MZ*mid* response. (C) The *mid* mutation perturbs activin-like signaling. MZ*oep* and MZ*mid* embryos were injected with RNA encoding either a *Xenopus* activin homologue (2.5 pg) or an activated form of mouse Smad2 (100 pg). Responses were assayed by observing *ntl* expression at 30–40% epiboly.

The presence of a notochord-like structure in MZ*sur* mutants suggests that these embryos retain some ability to transduce Nodal signals in a FoxH1-dependent manner. To test this idea, we mated rescued *sur* homozygotes with rescued *mid* homozygotes. Regardless of whether the male or female adult was the *sur* mutant, the progeny of these crosses largely resembled MZ*sur* embryos (Figure 3A). About 80% of these embryos (n = 375) display a morphological structure similar to the irregular notochords of MZ*sur* mutants, with the remainder lacking a recognizable midline structure. This result was also observed when MZ*mid* embryos were injected with RNA encoding the *sur* allele of *FoxH1* (80% MZ*sur* phenotype, n = 30). These observations support the hypothesis that the *sur* mutation of *FoxH1* represents a hypomorphic allele and not a null.

To directly test the ability of *FoxH1* mutants to transduce Nodal signals, we performed an overexpression assay using RNA encoding the Nodal homologue *squint* (*sqt*; Figure 3B). Wild-type and mutant embryos were injected with 50 pg of *sqt* RNA at the one-cell stage and fixed at 30–50% epiboly to examine their ability to upregulate expression of *ntl* and *gsc* in a Nodal-dependent manner. Wild-type embryos responded by ubiquitously activating both *ntl* (81/81) and *gsc* (85/87) expression. MZ*sur* embryos consistently upregulated both targets ubiquitously, similarly to wild-type (*ntl* 78/79; *gsc* 39/39). However, while *ntl* was activated throughout the animal pole in MZ*mid*, the activation was much weaker than in either wild-type or MZ*sur*, with the endogenous marginal expression pattern plainly visible (74/74). Upregulation of *gsc* was largely confined to the dorsal margin, where it is normally expressed, with little or no ventral or animal activation detected (32/37 dorsal expansion only, 4/37 weak animal activation, 1/37 no upregulation). These results suggest that while MZ*mid* mutants are able to respond to ectopic Nodal, presumably through at least one other transcription factor, the response is much weaker and/or restricted to endogenous marker expression domains.

MZ*oep* mutants, which lack the essential coreceptor of the Nodal pathway, have been reported to be entirely refractory to ectopically supplied Nodal. However, general Smad2/3-mediated TGFß signaling is attainable by bypassing the lack of the Nodal coreceptor. Injection of mRNA encoding either a *Xenopus* activin homologue (*XactßB*) or an activated truncation of mouse Smad2 (*mSmad2c*) into MZ*oep* causes robust activation of the Nodal targets *ntl* and *gsc*, as all intracellular components of the pathway are intact [3]. Because the *mid* allele encodes a putative truncated form of FoxH1 that lacks a SID, we hypothesized that treatments eliciting a Nodal-like response in MZ*oep* mutants would not be as effective in MZ*mid* embryos. Indeed, the lowest doses of *XactßB* (2.5 pg) and *mSmad2c* (100 pg) we found to produce a reliable upregulation of *ntl* in MZ*oep* mutants (66/71 and 8/21, respectively) had no effect in MZ*mid* mutants (0/21 and 0/68, respectively; Figure 3C). A higher dose of *XactßB* did produce a response, but a much weaker one than observed in MZ*oep* (data not shown). These results, together with the *sqt* injection data and the *sur*/*mid* trans-heterozygote phenotypes, indicate that *mid* encodes a stronger allele of *FoxH1* than *sur*, and that it causes deficiencies in general Smad2/3-mediated TGFß signaling.

The above findings support the notion that the *mid* allele behaves in a recessive fashion and is a stronger loss-of-function allele than *sur*. However, if the *mid* locus is producing a signal transduction-incompetent truncation of the FoxH1 protein, it may act as a recessive antimorph, potentially by blocking promoter binding by other transcription factors with which FoxH1 shares targets. To directly test this potential dominant inhibition by Mid protein, we injected *FoxH1*, *sur*, or *mid* mRNA into wild-type embryos to observe the resulting overexpression defects. Phenotypes were categorized as mild, severe, or catastrophic and included defects in anterior and axial structures. (see Table S1 for more detailed descriptions, and Figure S1 for examples of the defects observed). Injection of 50 pg *FoxH1* mRNA, five times the dose used to rescue *sur* or *mid* mutants, produced a range of defects, with only 25.6% of embryos (n = 297) appearing wild-type at 24 hpf. Injection of 50 pg *sur* mRNA yielded similar but somewhat stronger phenotypes, with only 17.6% of embryos (n = 136) appearing wild-type. In contrast, injection of 50 pg *mid* mRNA had a significantly weaker effect, with 85.6% of embryos (n = 167) appearing wild-type. These results suggest that, at identical doses, Mid protein overexpression has much less of an effect on development than either FoxH1 or Sur protein, and that physiological levels of Mid protein present in MZ*mid* mutants are most likely not acting in a dominant fashion.

We also wished to determine if blocking production of Mid protein in MZ*mid* mutants could alleviate any potential dominant effects of the mutant protein and produce embryos that resemble MZ*sur* mutants. Alternatively, if Sur protein retains some partial signaling capability, then preventing its production should abrogate this function and produce embryos that resemble MZ*mid* mutants. To block production of each of these mutant proteins, we injected MZ*sur* and MZ*mid* mutants with a morpholino targeting the translational start site of *FoxH1* mRNA (gift of B. Feldman; [17]). However, the published phenotype caused by this morpholino is more severe than either of the two maternal-zygotic *FoxH1* mutants. We therefore used a low dose of the morpholino to determine whether either of the mutants was sensitive to a partial depletion of mutant protein (Figure S2). Injection of 4 ng of *FoxH1*MO into MZ*sur* mutants caused a significant increase in embryos lacking a notochord (115/187 injected vs. 10/155 uninjected). Injection into MZ*mid* mutants never allowed formation of a structure resembling a notochord (0/182); in fact, a majority of these embryos (158/182) resembled their uninjected siblings. Together with the above overexpression data, these results strongly suggest that Mid protein does not act in a dominant fashion to disrupt axial development, and that Sur protein retains some capacity to transduce Nodal signals and allow for notochord formation.

### Mid and Sur proteins differ in their DNA–binding abilities

The difference in allelic strength between *mid* and *sur* seems to contradict the current consensus that *sur* represents a null allele of *FoxH1*. Due to the location of the *sur* lesion, which is either of two amino acid substitutions at the N-terminal extreme of the Forkhead DNA-binding domain, it had been assumed that *sur* caused a total loss of DNA binding, leading to a complete failure of signal transduction through Sur protein [15], [16]. This assumption is supported by a failure of the *sur* mutation to activate a luciferase reporter driven by an activin response element [17]. However, the highly penetrant losses of notochord markers and structure caused by the *mid* mutation, and the partial rescue of these axial phenotypes by providing the *sur* allele in *trans*, suggest that Sur protein retains some ability to transduce Nodal signals, albeit below wild-type levels. We therefore investigated the DNA-binding capabilities of the wild-type and mutant versions of zebrafish FoxH1 protein.

**Figure 4:**
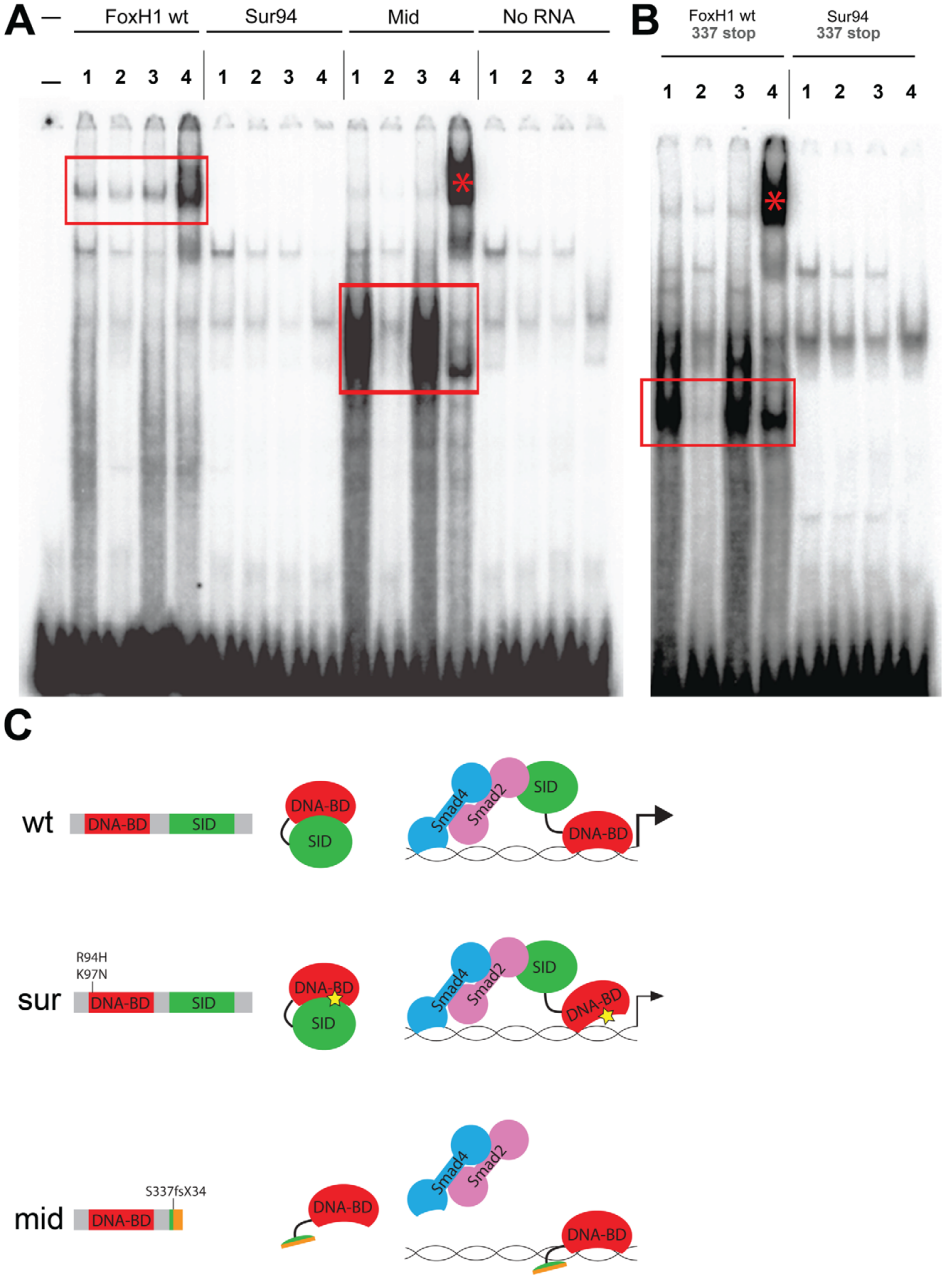
DNA–binding activities of FoxH1 mutants. (A) Electrophoretic mobility shift assays using a FoxH1 binding site probe derived from the zebrafish *gsc* promoter and *in vitro*-translated epitope-tagged full-length proteins. FoxH1 and Mid protein bind the probe in a protein- and sequence-specific manner (red boxes and asterisk), while Sur protein shows no binding activity. Upper lane labels indicate the RNA translated for use in each binding reaction; individual lane numbers denote additions to the basic binding reactions (1: no additions; 2: 100-fold excess unlabeled competitor probe; 3: 100-fold excess mutated unlabeled competitor probe; 4: anti-HA antibody). (B) EMSAs using truncated FoxH1 and Sur proteins lacking the C-terminal SID. Truncated wild-type protein specifically binds the probe (red box), while truncated Sur protein does not. Lane markings are as described in (A) above. (C) Model for DNA-binding activities of wild-type and mutant FoxH1 proteins. Wild-type protein binds weakly to its recognition sites alone, but can bind strongly upon loss of its C-terminus, suggesting that Smad interaction may “open up” the conformation of the wild-type protein and allow for strong binding upon pathway activation. Sur protein is impaired in its DNA-binding ability, but may be weakly/transiently tethered to its recognition sequences by activated Smads or other unknown factors. Mid protein cannot interact with Smads and so cannot transduce Nodal signals, but can bind strongly to FoxH1 recognition sites.

We employed an electrophoretic mobility shift assay using a DNA probe derived from a putative FoxH1 binding site in the proximal promoter of the zebrafish *gsc* gene and *in-vitro* translated FoxH1, Sur, and Mid proteins N-terminally tagged with 6xHis and 3xHA epitopes (Figure 4A). Wild-type FoxH1 protein shifted the probe in a protein- and sequence-specific manner, as judged by effective competition by unlabeled probe, no competition by unlabeled mutated probe, and successful supershifting by an antibody against the HA epitope. However, Sur protein produced no detectable shift of the probe, suggesting that the *sur* mutation does indeed abolish DNA binding. Surprisingly, a qualitative analysis suggests that Mid protein binds to the probe much more strongly than wild-type protein. Because the *mid* mutation removes the C-terminal 25% of the FoxH1 polypeptide, we speculated that, in the absence of activated Smads in the nucleus, the C-terminus of wild-type FoxH1 may normally function to occlude the DNA-binding domain. If so, we hypothesized that the complete loss of binding we observed for the Sur protein may actually be a combination of two separate phenomena: a partial impairment in DNA binding caused by the *sur* mutation, and the wild-type C-terminal occlusion of the DNA-binding domain. Removal of the C-terminus from the Sur polypeptide may then reveal a weak DNA-binding ability of the Sur DNA-binding domain.

To determine whether the C-terminus of Sur protein was masking some partial ability of the mutated Forkhead domain to bind to its DNA recognition sequence, we generated C-terminally truncated versions of wild-type FoxH1 and Sur proteins (denoted by “337 stop”). We observed that the wild-type truncated protein now bound to the probe much more strongly than full-length FoxH1 and at a level comparable to Mid protein, supporting the idea that the C-terminus possesses some ability to partially inhibit DNA-binding by the Forkhead domain (Figure 4B). However, the truncated Sur protein still did not shift the probe, indicating that the *sur* mutation genuinely abolishes DNA-binding, even without a potential inhibitory activity from the C-terminus. This result is surprising given the weaker phenotypes and stronger ectopic-Nodal responses of MZ*sur* compared to MZ*mid*. However, it may be possible that interaction with activated Smads is enough to weakly tether the Sur protein to its target promoters long enough to activate some transcription (Figure 4C).

### FoxH1 and Bon do not account for all zebrafish Nodal transduction

**Figure 5:**
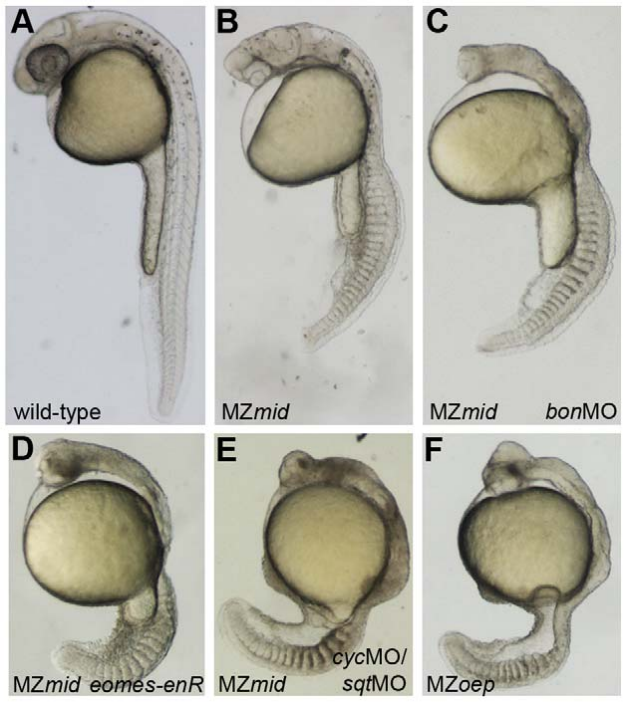
Inhibiting Nodal signals in MZ*mid* mutants. Wild-type (A), uninjected MZ*mid* (B), and injected MZ*mid* (C–E) embryos compared to a complete loss of Nodal signaling in MZ*oep* (F) mutants. Inhibiting *bon* function (3 ng *bon*MO) in an MZ*mid* background further impairs anterior development but does not affect tail development (C). Blocking *eomes* function (15–20 pg *eomes-enR* mRNA; D) resembles both a loss of the Nodal ligands *cyc* and *sqt* (8 ng each *cyc*MO+*sqt*MO; E) and MZ*oep* (F), indicating that Nodal signaling is occurring in MZ*mid* mutants and is mediated by Eomes.

Since *mid* phenotypes more closely resemble those of FoxH1 loss in other organisms, we used MZ*mid* and MZ*oep* mutants as representatives to study the differences between loss of FoxH1 and loss of Nodal across species. MZ*mid* embryos clearly develop more somites and are generally larger than MZ*oep* mutants, and form a clear mid-hindbrain boundary (Figure 5B, 5F). Furthermore, early markers of endoderm specification that are lost in MZ*oep* mutants are expressed in MZ*mid* embryos: while significantly reduced, *bon*, the zebrafish Mixer homologue, is expressed prior to gastrulation (70/75), and *casanova/sox32* (*cas*; n = 38), *axial* (n = 46), and *sox17* (n = 34) are expressed abundantly in endoderm precursors of all late-gastrulation MZ*mid* mutants analyzed (Figure 2L, 2R and data not shown). Because the *oep* mutation disrupts the Nodal pathway at a very early step in signal transduction (ligand-receptor binding) whereas the *mid* mutation affects a later step (transcriptional target activation), we wanted to confirm that the phenotypic differences between these two mutants was due to an otherwise intact Nodal pathway in MZ*mid* embryos. We injected MZ*mid* embryos with a mixture of morpholinos targeting the Nodal homologues *sqt* and *cyclops* (*cyc*) [24], which effectively phenocopy the defects of both MZ*oep* mutants and *cyc*;*sqt* double mutants [3]. Knockdown of these Nodal homologues in MZ*mid* efficiently phenocopies the MZ*oep* defects (139/139), indicating that Nodal signals are active in MZ*mid* (Figure 5E). Therefore, we hypothesized that at least one other transcription factor capable of transducing Nodal signals, presumably through interaction with activated Smad2/3, was present in MZ*mid* embryos.

Mixer/Bon has previously been proposed as a compensatory factor upon loss of FoxH1 function in MZ*sur* mutants [18]. Bon was already known to be very important for proper endoderm specification in a Nodal-dependent manner [25], and could physically interact with phosphorylated Smad2 as part of the Nodal pathway [26]. However, loss of Bon function in MZ*sur* mutants did not recapitulate the MZ*oep* phenotype, leading to the assumption that another transcription factor was transducing Nodal signals in these embryos [18]. Since our results suggest that *sur* does not completely abolish FoxH1 function, it is possible that this previous study did not involve a complete loss of Nodal signaling through FoxH1 and Bon together. We therefore injected a morpholino targeting *bon* into MZ*mid* mutants to attempt to phenocopy the MZ*oep* phenotype. At a dose that reliably knocked down endoderm marker expression in wild-type embryos (data not shown), *bon*MO caused a further impairment in anterior neural patterning in MZ*mid* mutants, as judged by the loss of a morphological mid-hindbrain barrier and a striking similarity to the anterior structures of MZ*oep* embryos (77/79; Figure 5C). This effect was somewhat expected, given a prior study implicating *bon* in organizer specification, prechordal plate formation, and proper neural patterning [27]. However, outside of the head region, *bon*MO did not have much of an effect on a gross morphological level (77/79). The embryos developed the same numbers of somites and were generally the same size as their uninjected siblings. Therefore, in agreement with the aforementioned investigation into the interaction between the *sur* and *bon* mutations, we conclude that FoxH1 and Bon do not represent the entire complement of Nodal-transducing transcription factors in the early zebrafish embryo.

### Eomes inhibition in MZ*mid* recapitulates a total loss of Nodal signaling

An intriguing candidate for another Nodal responsive factor is Eomesodermin (Eomes), a T-box transcription factor repeatedly implicated in mesendoderm induction in several species. In *Xenopus*, Eomes is one of the earliest expressed mesoderm inducers, and its overexpression leads to ectopic activation of a number of Nodal signaling targets, such as *Xbra* (the *Xenopus* homologue of *no tail*) and *gsc*. Inhibition of Eomes causes gastrulation failure and mesoderm marker downregulation [28]. A more recent study showed that cooperative Nodal signaling and Eomesodermin function are required during *Xenopus* paraxial mesoderm induction [29]. In mouse, Eomes is required for proper prospective mesoderm ingression through the primitive streak and the consequent formation of the mesoderm germ layer [30], and also for the definitive endoderm lineage [31]. Zebrafish Eomes has similarly been implicated in the Nodal-dependent induction of dorsal mesoderm markers [32] and is required for endoderm specification [33]. Recently, *Xenopus* Eomes protein was shown to physically interact with phosphorylated Smad2, potentially placing it parallel to FoxH1 in the Nodal signaling pathway [34]. Eomes was therefore a prime candidate for mediating the Nodal-dependent mesendoderm induction observed in MZ*mid* mutants.

In order to test this hypothesis, we wanted to inhibit Eomes function in an MZ*mid* background. However, *eomes* morpholinos have limited effects on development, most likely due to the presence of maternally deposited Eomes protein in zebrafish oocytes [32]. We therefore employed a fusion of the DNA-binding domain of zebrafish EomesA and the transcriptional repressor domain of the *Drosophila* Engrailed protein (*eomes-enR*; gift of A. Bruce) in order to block endogenous Eomes function. This fusion was shown previously to inhibit dorsal mesoderm marker expression, and its effects on development could be rescued by coinjection of *eomesA* mRNA [32]. When injected into MZ*mid* mutants at the one-cell stage, *eomes-enR* caused an impairment of anterior neural development similar to inhibition of *bon* function (78/122; Figure 5D). However, in contrast to *bon*MO, which had no discernible effect outside of the head region, injection of *eomes-enR* also caused a significant reduction in embryo length with a coincident decrease in somite number. These embryos closely resembled the MZ*oep* phenotype at 24 hpf (84/122), suggesting that Eomes is, in fact, responsible for Nodal signaling functions observed in MZ*mid* mutants.

**Figure 6:**
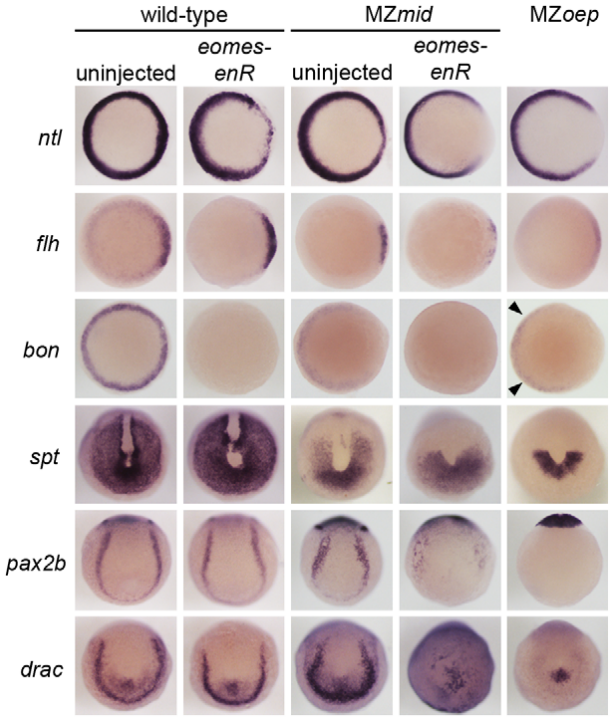
Eomes inhibition enhances the Nodal signaling and mesendoderm deficiencies of MZ*mid*. Markers for various populations of mesendoderm derivatives were analyzed in wild-type and MZ*mid* embryos in which Eomes function was inhibited. Eomes inhibition in wild-type embryos has a minimal effect on expression of most markers, whereas in an MZ*mid* background it has significant effects on mesendoderm-derived tissues, approaching MZ*oep* levels for most markers. Arrowheads indicate Nodal-independent expression of *bon* in MZ*oep* mutants.

To validate this result, we examined expression of a number of mesendoderm markers in MZ*mid* mutants injected with *eomes-enR* (Figure 6). Repression of Eomes function in an MZ*mid* background appears to exacerbate the preexisting dorsal mesoderm marker reduction at 50% epiboly. Residual dorsal *ntl* and *flh* expression in *MZmid* (38/38 and 38/38, respectively) were further downregulated by *eomes-enR* (51/51 and 47/51, respectively), whereas expression of these markers in wild-type embryos was only mildly affected, if at all, upon Eomes inhibition alone (11/54 and 7/33 with mild reductions, respectively). Intriguingly, blocking Eomes function in both wild-type (56/56) and MZ*mid* (53/53) embryos strongly reduces or completely abolishes *bon* expression, whereas MZ*oep* mutants exhibit very weak expression of *bon* prior to gastrulation (30/35; arrowheads in Figure 6), as has been reported previously [18], [35]. This result indicates that Eomes is required to initiate *bon* expression and may do so in a Nodal-independent manner. Importantly, we observed that repression of Eomes function did not affect the residual expression of *cyc* or *sqt* in MZ*mid* mutants (Figure S3). All MZ*mid* mutants analyzed showed marginal expression of *cyc* (59/59) and *sqt* (53/53) at 30–40% epiboly, and injection of the *eomes-enR* mRNA into MZmid did not perturb expression of either *Nodal* homologue (70/70 and 54/54 with unaffected *cyc* and *sqt* expression, respectively). While these results do not address a potential ability of Eomes to regulate *Nodal* expression during later stages of development, they indicate that Eomes is not involved in regulation of *cyc* or *sqt* during Nodal-dependent mesendoderm induction, as FoxH1 is [10], [15], [36]–[38].

Because Eomes can regulate certain Nodal transcriptional targets, we investigated whether FoxH1 and Eomes shared roles in the morphological development of dorsal mesoderm-derived structures. First, MZ*mid* mutants were injected with mRNA encoding a translational fusion of the Eomes DNA-binding domain and the VP16 transcriptional activator domain (gift of A. Bruce, [32]). This fusion was previously shown to upregulate several Nodal targets in a Nodal-dependent manner. When injected into MZ*mid* mutants, *eomes-VP16* caused a significant rescue of notochord development (Figure S4A; 102/201). While this result suggests that FoxH1 and Eomes both contribute to notochord development, it should be noted that this effect was observed upon ubiquitous overexpression of a constitutively active form of Eomes. To test whether Eomes normally contributes to notochord development, we injected wild-type embryos with *eomes-enR*. Importantly, we very rarely observed a loss of notochord in wild-type embryos injected with *eomes-enR* (Figure S4B; 2/156 with no notochord), although these embryos do display other severe morphological defects. This result demonstrates a certain level of specificity of the Eomes fusion proteins, as other T-box functions, like that of *no tail* in notochord development [39], are not disrupted by *eomes-enR*. It also suggests that properly regulated Eomes protein, expressed at endogenous locations and levels, does not play a significant role in notochord specification.

In addition to dorsal mesoderm and endoderm induction, which are well-known Nodal-dependent processes, other mesoderm-derived tissues present in *MZmid* but reduced or absent in *MZoep* are affected by Eomes repression (Figure 6). Because we observed a reduction in somite number in MZ*mid* embryos injected with *eomes-enR*, we examined the expression of *tbx16*/*spadetail* (*spt*), a T-box factor known to be required for proper somitogenesis, especially for formation of the anterior (trunk) somites [40]. At the end of gastrulation, *spt* is expressed in the presomitic mesoderm at the vegetal pole of the embryo and excluded from the dorsal axis. MZ*mid* mutants display a partial reduction in the *spt* expression domain, which resembles a vegetal “U” with expression reaching into the dorsal side of the embryo (53/53). However, *eomes-enR* further restricted the expression domain of *spt* (40/52) to one resembling that observed in MZ*oep* embryos, which express *spt* only in a semicircular band of cells at the ventrovegetal extreme of the embryo (36/36). This further reduction of *spt* expression likely explains the significant loss of anterior somites observed later in MZ*mid* mutants injected with *eomes-enR*. A similar reduction of expression is observed for *pax2.1* (56/62) and *draculin* (39/39), markers of the intermediate mesoderm and blood precursors, respectively, which appear wild-type in uninjected MZ*mid* embryos (65/65 wild-type for *pax2b*; 36/36 wild-type for *draculin*). While Eomes inhibition in wild-type embryos had little effect on expression of these markers (78/78 wild-type for *pax2b*; 47/47 wild-type for *draculin*), it drastically reduced their expression in an MZ*mid* background. Based on the morphological and marker expression phenotypes in MZ*mid* mutants upon inhibition of Eomes function, we propose that Eomes is the transcription factor responsible for the Nodal-dependent mesendoderm specification observed in embryos lacking Nodal transduction through FoxH1.

## Discussion

### Transcriptional regulation of Nodal signaling

**Figure 7:**
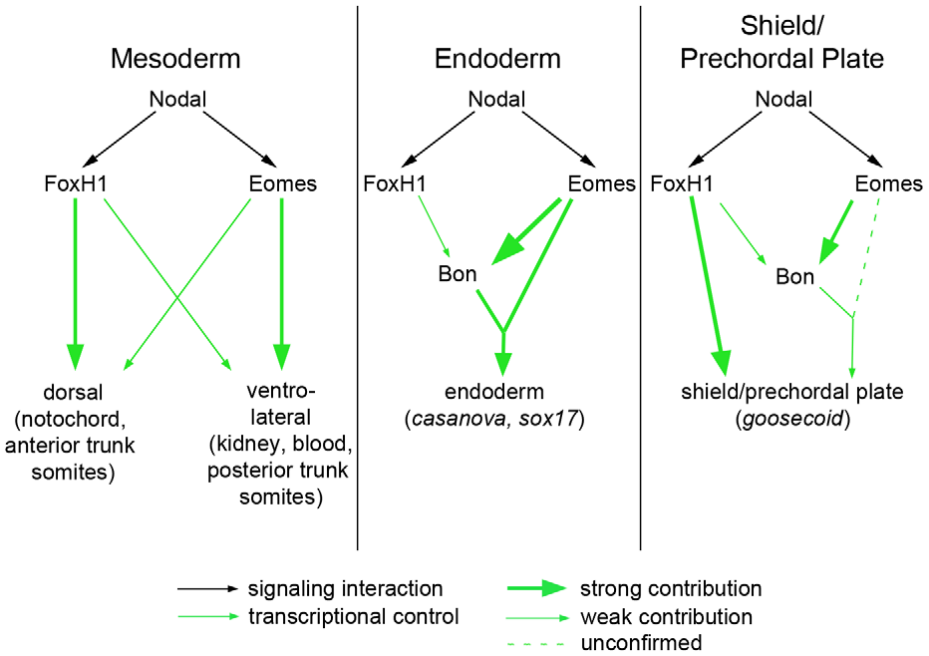
Model for roles of Nodal signaling in early zebrafish embryos. Nodal signaling functions are partially separable according to the transcription factors used by the pathway. (A) FoxH1 is absolutely required for notochord specification and for the most anterior trunk somites, while Eomes is essential for nonaxial mesoderm induction upon loss of FoxH1 function. These two factors may cooperate in a wild-type embryo for normal nonaxial mesoderm induction, as loss of either alone does not have a significant impact on early marker expression (Figure 6). (B) FoxH1 is not strictly required for the endoderm specification pathway, although it makes contributions to *bon* expression. Eomes is required both for *bon* initiation and for proper downstream endoderm marker expression, as has been demonstrated previously. (C) In the organizer, FoxH1, Eomes, and Bon function meet for full induction of marker expression and prechordal plate derivation.

The ability of many intercellular signaling pathways to function effectively depends on the transcription factors that regulate target gene expression. Our investigation into the early roles of Nodal signaling reveals distinct and biologically separable functions for the Nodal pathway during zebrafish development, contrary to the current understanding of Nodal-dependent mesendoderm specification. Through identifying a novel mutation in the Nodal signaling effector FoxH1 that removes its ability to bind to the Smad intracellular Nodal effectors, we have shown that certain functions of the pathway can be blocked without overtly perturbing its other roles. This functional segregation is achieved, at least in part, by the use of multiple transcription factors capable of interacting with Smad2 upon stimulation of the pathway (Figure 7). FoxH1 appears to be absolutely required for formation of the notochord and the most anterior trunk somites. Eomesodermin is responsible for much of the nonaxial mesoderm specification observed in MZ*mid* mutants, although in wild-type embryos Eomes and FoxH1 cooperate in this function, since loss of either protein alone does not appreciably affect most nonaxial mesoderm marker expression. Eomes is also required for at least two steps of endoderm specification: the initiation of *bon* expression (Figure 6) and the assembly of a transcriptional activator complex, composed of Eomes, Bon, and Gata5, on the *cas* promoter [33]. Despite these partially separable roles of the Nodal responsive transcription factors, their activities appear to converge at the shield/organizer, which will later form the prechordal plate. All three proteins contribute to marker expression at the shield and to subsequent function of the prechordal plate in neural development, based on the loss of morphological neural structures (such as the mid-hindbrain boundary) in pairwise versus single loss of function situations (Figure 5). It is unclear whether Eomes' contribution to prechordal plate specification derives from direct target activation or through its activation of Bon. However, given the relatively minor neural patterning and morphology defects in *bon* mutants [27], and Eomes' reported ability to induce entire secondary axes when overexpressed in zebrafish [32], it is highly likely that it is making direct contributions to shield formation and function.

### DNA–binding capabilities of wild-type and mutant FoxH1 proteins

We have also demonstrated that the previously characterized *sur* allele is not a complete loss of function, contrary to current assumptions. Based on the two mutants' phenotypes and relative abilities to transduce Nodal signals, we find that *sur* retains some *in vivo* activity and therefore represents a less deleterious mutation of *FoxH1* than *mid*. Surprisingly, despite the evidence supporting *sur*'s partial functionality, we find that the *sur* mutation genuinely abolishes binding to a target gene reporter element in a gel shift assay. If Sur protein cannot bind its target regulatory sequences in the genome, how can it activate target genes in response to Nodal? One possibility is that its interaction with activated Smads in the nucleus is enough to allow it to transiently interact with its target sequences through the weak DNA-binding ability of Smad4 (Figure 4C). In a similar vein, it should be noted that the broader environment of FoxH1 binding sites in the genome has not been well studied; there may be other factors with which FoxH1 normally interacts at its target sites to efficiently activate transcription *in vivo*. Sur protein may still be able to interact with these hypothetical factors, allowing for weak tethering of an otherwise active Sur-Smad complex to its genomic binding sites.

The observation that *in vitro* translated Mid protein can bind to a FoxH1 target probe, and with apparently greater affinity than full-length FoxH1, might suggest that the Mid protein could possess dominant negative or recessive antimorphic functions. A mutated transcription factor with tighter binding to its consensus site that is unable to carry out target gene transcription could theoretically compete with wild-type protein for consensus occupation. However, several of our findings indicate this is not the case. *mid* heterozygous fish are viable and produce phenotypically mutant progeny at a typical Mendelian frequency, and MZ*mid* defects can be partially rescued by *sur* RNA injection. Furthermore, overexpression of *mid* mRNA results in developmental defects much less frequently than either *FoxH1* or *sur* RNA, and morpholino knockdown of any endogenous Mid protein in MZ*mid* mutants does not rescue notochord formation. Finally, we do not see alterations in the expression domains of certain genes whose promoters are bound by the FoxH1 protein, including *cyc*, *sqt*, and nonaxial *ntl*, arguing that the mutant FoxH1 protein in *midway* is not blocking these promoters. Given these results, dominant-negative and recessive antimorphic activities for Mid protein seem highly unlikely. Indeed, documented instances of inhibitory functions for FoxH1 involve recruitment of co-inhibitors. The Forkhead domain of mouse Foxh1 recruits Gsc to the murine *Mixl1* promoter, allowing for proper histone deacetylase-mediated repression of the *Mixl1* locus during early development [41]. Foxh1 can also inhibit the transcription of *gsc* itself by incorporating Smad3, rather than Smad2, into a regulatory complex at the murine *gsc* promoter in luciferase assays [42]. Therefore, it is possible that the mere presence of non-functional Mid protein at a FoxH1 target promoter does not strictly imply inhibition of that promoter. It seems more likely, based on our *in vivo* results, that an activated FoxH1-Smad2/4 complex has a higher affinity for its target promoters than do the truncated Mid protein or the full-length wild-type FoxH1 protein alone.

Our investigation into the biochemical properties of the wild-type and mutant FoxH1 proteins revealed a new potential regulatory mechanism for FoxH1 transcriptional activity. Removal of the C-terminal Smad interaction domain, either through artificial truncation or through the *midway* frameshift, allows qualitatively enhanced DNA binding of the truncated protein relative to the full-length wild-type protein. This apparent autoinhibitory function of the FoxH1 C-terminus is not an unprecedented phenomenon. Several transcription factors display autoinhibition activities between their DNA-binding domains and other regions of their polypeptides, including the Ets-1 protooncoprotein [43]–[46] and the cell-cycle transcription factor Swi4 [47]. In fact, the mechanism of Swi4 autoinhibition relief may be similar to what we propose for FoxH1. C-terminal occlusion of the Swi4 DNA binding domain is interrupted by interaction with Swi6, much as Smad2/4 binding to the FoxH1 C-terminus may serve to “open up” the polypeptide to reveal the Forkhead DNA-binding domain. A detailed structural and biochemical analysis of this autoinhibition activity will shed light on this potential mode of FoxH1 transcriptional regulation.

### Potential Nodal-independent roles for FoxH1 in early development

A recent study investigated the functions of FoxH1 in early zebrafish development by use of morpholinos to knock down *FoxH1* translation [17]. Their results indicated a very early, and potentially Nodal-independent, role for FoxH1 during epiboly. High doses of a *FoxH1* translation-blocking morpholino caused embryos to developmentally freeze at about 50% epiboly, remaining temporally halted while their control-injected siblings progressed relatively normally through gastrulation and somitogenesis. The highly penetrant MZ*mid* phenotype we have described here, which closely resembles loss of *FoxH1* function in other organisms, is not nearly as severe as that of *FoxH1* morphants. However, while the *mid* mutation removes FoxH1's ability to transduce Nodal signals via Smad interaction, it is possible that the Forkhead DNA-binding domain retains some function in MZ*mid* embryos that is lost in the *FoxH1* morphants. Indeed, certain studies have described Nodal-independent functions for the FoxH1 Forkead domain in other organisms. During anterior heart field development in mouse, the Forkhead domain of FoxH1 was shown to bind to Nkx2.5, and this interaction was required to fully activate a *mef2c* transcriptional reporter [48]. In light of this observation, and the aforementioned interaction with Gsc at the mouse *Mixl1* promoter, it is possible that the Forkhead DNA-binding domain of FoxH1 retains some early developmental functions in MZ*mid* embryos that are blocked by morpholino knockdown. Indeed, we occasionally observed a midline bifurcation phenotype in MZ*mid* embryos injected with a low dose of *FoxH1* MO (4/182). This defect is reminiscent of the low-penetrance midline bifurcation phenotype observed in MZ*sqt* embryos [49] and ethanol-induced midline bifurcations in wild-type zebrafish embryos [50], both of which are attributed to impairments in gastrulation movements during early development. Given the Nodal-independent inhibition of cell motility described in the original *FoxH1*MO study, it seems likely that the Mid protein (i.e. the FoxH1 DNA-binding domain) can still act to promote cellular movements at the start of gastrulation. In further support of the Mid protein having some functionality, we also observed a few MZ*mid*;*FoxH1*MO embryos that developed two eyes (5/182; see Figure S2 caption), a phenotype we never see in MZ*mid* mutants. Therefore, we postulate that the Mid protein can still function endogenously to repress some targets that affect prechordal plate formation. In fact, it is known that mouse Foxh1, in cooperation with Gsc, can repress *mixl1* (the mouse *bon* homologue). Normally this repression would occur simultaneously *with* strong Nodal signaling to allow for prechordal plate formation. It is intriguing to speculate that the Mid protein still binds to and represses this promoter in zebrafish, in cooperation with the small amount of Gsc being produced, and that this repression is not overcome since Nodal signaling is impaired. Upon *FoxH1*MO injection into MZ*mid* mutants, this endogenous function of the FoxH1 DNA-binding domain is blocked, allowing for enough prechordal plate function to split the eye field on rare occasions. For these reasons, we believe that the *mid* allele is not a complete *FoxH1* null mutation, but one that eliminates a specific function of the protein (the Smad-mediated Nodal signal transduction) while leaving other endogenous functions intact. Investigation into the phenotypic differences between MZ*mid* mutants and *FoxH1* morphants could uncover further novel roles for this transcription factor during early development. Thus, the *midway* mutation will serve as an important tool for understanding the Nodal dependent and independent roles of FoxH1 in development.

### Evolution of the notochord specification program

The strict requirement of FoxH1 for notochord development, as observed in MZ*mid* mutants, may shed some light on a long-standing evolutionary question: how did the notochord genetic program evolve? Homologues of many known players in notochord specification and development have been identified outside of the chordate lineage. Nodal pathway components are found in non-chordate deuterostomes [51], [52] and non-deuterostome bilaterians [53], and homologues of *brachyury/ntl* have been found in protostomes [54] and possibly in the last common ancestor of bilateria and sponges [55], [56]. Therefore, these factors alone cannot account for the evolution of the notochord, indicating that other evolutionary innovations must have arisen in order to harness this machinery for use in notochord development. Intriguingly, although the Forkhead superfamily is represented in all animal phyla [56] and even in fungi [57], members of the FoxH subfamily have not been identified outside of the chordate subgroups of the deuterostome lineage [56], [58]. FoxH homologues have been discovered in the tunicate *Ciona intestinalis* [58] and the cephalochordate *Amphioxus* [59], but are absent in echinoderm genomes [52], [60], suggesting that this Forkhead subfamily could have evolved concomitantly, and perhaps causally, with the chordate lineage. Indeed, a recent analysis of the regulation of “notochord-specific” genes in *Ciona* indicates that a cohort of these genes falls outside the transcriptional control of Brachyury [61]. It is tempting to consider that some of these genes, which may be essential for the formation of the notochord in *Ciona* and chordates, could be transcriptionally regulated by FoxH homologues.

This proposed recent evolution of the FoxH subfamily may help explain an apparent discrepancy between the MZ*mid* phenotypes and the current model for Nodal-mediated mesendoderm induction. A host of experiments in various species [62] indicates that high levels of Nodal signals are required for endoderm and prechordal plate specification, tissues which are most sensitive to partial reduction of Nodal signaling. Lower levels are sufficient for specification of dorsal mesoderm fates such as notochord, which are relatively unperturbed upon partial loss of Nodal signals. In conflict with this model, MZ*mid* embryos consistently lack notochords, as is seen in *Foxh1* knockout mice [8], [10], while prechordal plate expression of *gsc* is reduced but present and early endoderm specification appears largely unaffected. If FoxH1 evolved relatively recently in deuterostomes, its unique function in notochord formation would have been superimposed upon the preexisting dose-dependent functions of the Nodal pathway in the differential specification of mesendoderm. Perhaps this late addition of the notochord genetic program causes it to fall outside of the Nodal dose-response model of mesendoderm induction. In chordates, then, endoderm and nonaxial mesoderm would be induced by higher and lower levels of Nodal signals, respectively, with FoxH1 serving mainly to properly modulate expression levels via Nodal feedback loops. The newly evolved axial mesoderm domain, however, would form through direct activation by FoxH1 of a notochord-specific gene network in a particular location of the embryo, relying on this specific activity of FoxH1 rather than the preexisting Nodal dose-response mechanism. This evolutionary layering of Nodal signaling responses in chordates may have been further complicated by divergences among the different vertebrate lineages, as loss of Foxh1 function in mice does lead to a failure of definitive endoderm induction [8], [10].

In sum, our current work demonstrates that FoxH1 plays a required conserved role in notochord specification through characterization of the novel *FoxH1* mutant *mid*. We also identify Eomes as a Nodal-transducing factor that acts, directly and indirectly, in concert with FoxH1 to carry out all Nodal-dependent processes during early mesendoderm specification. Our results provide novel insights into the previously unappreciated separation of roles of the Nodal pathway based on the transcription factors available for signal transduction. We also shed light on the evolution of the genetic program that leads to development of the chordate animal lineage.

## Materials and Methods

### Ethics statement

All protocols for the care and use of zebrafish were approved by Princeton's Institutional Animal Use and Care Committee and the University Veterinarian.

### Zebrafish strains

*FoxH1^m768^* and *oep^tz257^* were generated in the Boston and Tübingen ENU mutagenesis screens, respectively [63], [64]. *FoxH1^Pr1^* was isolated as a spontaneous mutation by R. Burdine. Fish strains were maintained by outcrossing to various wild-type strains, including AB and WIK, and recessive pigment mutants including *alb*, *gol*, and *leo*.

### Mapping, sequencing, and genotyping the *midway* locus

The *mid* locus was mapped to a 10 cM region of chromosome 12, first using a genome-wide panel of SSLP markers for low-resolution mapping and then using additional selected SSLP markers for finer mapping [23]. This region, defined by markers z27025 and z11549, covered the *FoxH1* locus, prompting complementation matings between *sur* and *mid* heterozygotes. Upon complementation failure, the *FoxH1* locus was sequenced in *mid* homozygotes, and a two-nucleotide insertion was discovered after nucleotide 1007 of the open reading frame.

A three-nucleotide deletion in the *mid* 3′UTR was used as a RFLP for genotyping purposes. A 324-bp region was amplified using the following primers: forward 5′- CCAGTATGCCCTACAGAACGGACCTTCCC-3′; reverse 5′-CTGTACAACAGCTTGTTGCCAGGGC-3′. This product was digested with BtsCI, which cuts only the mutant fragment to produce a 200-bp band upon 3% agarose gel electrophoresis.

### Plasmid construction

The complete *FoxH1* cDNA clone was obtained from American Type Culture Collection (GenBank ID BC044340.1). The *FoxH1* cDNA was subcloned into pCS2+ using the flanking *XhoI* sites to make the pCS2-*FoxH1* expression plasmid. For the related *sur* and *mid* expression plasmids, the *sur^m768^* and *mid* mutations were engineered into pCS2-*FoxH1* using the Stratagene QuikChange II Site-Directed Mutagenesis Kit (Agilent #200523) and verified by sequencing. To make the epitope-tagged expression constructs for EMSAs, the *FoxH1*, *sur*, and *mid* ORFs were PCR-amplified from the pCS2 expression vectors and ligated into a modified pET15b vector containing an expanded multiple cloning site and three copies of the hemagglutinin epitope tag in-frame downstream of the 6xHis sequence. The entire 6xHis-3HA-*FoxH1* cassette of each pET15b plasmid was then PCR-amplified and ligated into pCS2. The *mid* ORF contained the entire *FoxH1* ORF with the *mid* AT insertion; plasmids containing this ORF produced a polypeptide that was of the predicted smaller size compared to the wild-type and *sur* plasmids upon *in vitro* transcription/translation and Western blotting. Truncated FoxH1 and Sur expression plasmids were created by PCR-amplifying the corresponding ORFs from the full-length constructs using a reverse primer that replaced the serine at codon 337 with a stop codon (e.g. FoxH1-337X).

### Whole-mount *in situ* hybridization

Antisense RNA probes were transcribed from linearized plasmid templates using DIG-labeled nucleotides and used in a standard protocol for whole-mount *in situ* hybridization [65]. Probes used were *no tail* [66], *goosecoid* [67], *bonnie and clyde/mixer* [68], *axial/FoxA2/HNF3ß* [69], *casanova/sox32* [70], *floating head/Znot* [71], *spadetail/tbx16* [40], *pax2.1* [72], *draculin* [73], *sonic hedgehog* [74], *lefty1/antivin* [75], *squint/ndr1* [2], [76], *cyclops/ndr2* [77], southpaw/*ndr3* [19], *sox17* [35], and *collagen2a* [78].

### Microinjections

*In vitro*-transcribed mRNAs were generated from linearized plasmid templates using the mMessage mMachine SP6 transcription kit (Ambion #AM1340). Template plasmids were: pCS2-*FoxH1*, pCS2-*sur*, pCS2-*mid*, pCS2-6xHis-3HA-*FoxH1*, pCS2-6xHis-3HA-*sur* (see Plasmid Construction above), pCS2-*squint* [2], pSP64T-*XactßB* [79], pCS-cytßgal-*madr2*(C) [80], and pENG-N-*eomes* and pVP16-N-*eomes* [32]. *sur* and *mid* homozygous embryos were rescued by injection of 10 pg of *FoxH1* mRNA transcribed from pCS2-*FoxH1*, raised to adulthood, and genotyped as described above. Morpholinos for *FoxH1*, *bonnie and clyde*, *squint*, and *cyclops* were described previously [17], [18], [24], [27], [81], [82]. mRNAs and morpholinos were diluted in 10 mg/mL Phenol Red and injected in 500 pL drops into the yolks of 1–4 cell stage embryos.

### Microscopy

Live embryo and *in situ* hybridization images were captured at 4× or 10× magnification using a ProgressC14 digital camera (Jenoptik) on a Leica MZFLIII microscope.

### Electrophoretic mobility shift assays

Probe synthesis and labeling were performed as described [83]. The double-stranded FoxH1 binding site probe was derived from a 36-base pair sequence centered around a putative FoxH1 binding site in the zebrafish *goosecoid* proximal promoter (wild-type: 5′-TCAAATTAATTCTCAATACACAGATCGGTGGTTTTC-3′; mutant: 5′-TCAAATTAATTCTCAAGACCCAGATCGGTGGTTTTC-3′; underlined bases denote FoxH1 binding site).

pCS2-6xHis-3HA plasmids (see Plasmid construction above) were linearized with Asp718I and transcribed using SP6 RNA polymerase and a ribonucleotide mixture containing 7-methyl guanosine. mRNAs were subsequently used in *in vitro* translation reactions using a rabbit reticulocyte lysate system (Promega). Relative amounts of translated protein in each reaction were determined by Western blotting for the HA epitopes, and equal amounts of proteins were used in binding reactions as described [83] with the addition of 50 ng/µL each of poly(dI-dC)/poly(dI-dC) and poly(dA-dT)/poly(dA-dT).

## Supporting Information

Figure S1 Defects caused by overexpression of *FoxH1*, *sur*, and *mid* mRNA. Representative images of common phenotypes observed upon *FoxH1*, *sur*, and *mid* overexpression in wild-type embryos. (A) Embryo injected with 100 pg *mid* mRNA exhibiting a wild-type appearance at 24 hpf. (B) Embryo injected with 100 pg *mid* mRNA exhibiting a wavy notochord. (C) Embryo injected with 50 pg *FoxH1* mRNA exhibiting a loss of eyes and head structures, and a morphologically irregular notochord (also see panel F). (D) Dorsal anterior view of an embryo injected with 50 pg *sur* mRNA exhibiting eyes of unequal sizes. (E) Dorsal anterior view of an embryo injected with 50 pg *FoxH1* mRNA exhibiting a single unilateral eye. (F) Enlarged portion of embryo in panel C at the level of the yolk extension. Arrows indicate the abnormal notochord.
(TIF)

Figure S2 Effects of *FoxH1* knockdown on MZ*sur* and MZ*mid* mutants. MZ*sur* and MZ*mid* embryos were injected with 4 ng FoxH1MO to assess the effect of inhibiting production of mutant FoxH1 proteins on development. A majority of injected MZ*sur* embryos lack notochords (115/187 without notochords); injection into MZ*mid* mutants never rescues notochord formation (0/182 with notochords). 15/182 injected MZmid mutants had slight defects including delayed development and nonspecific necrosis which may be injection artifacts. 4/182 displayed midline bifurcations. In 5/182 embryos, injection of FoxH1MO into MZ*mid* caused splitting of the normally fused eye field into two eyes. This effect is most likely related to the published role of the Foxh1 DNA-binding domain in inhibiting the *mixl1* promoter via recruitment of Gsc protein in mouse _ [41] (see Discussion for more details).
(TIF)

Figure S3 *Nodal* ligand expression in MZ*mid* mutants Pre-gastrulation expression of *cyc* and *sqt* were analyzed in uninjected and *eomes-enR*-injected MZ*mid* embryos. Expression resembles that in wild-type embryos, though at lower levels, and is unaffected by Eomes inhibition.
(TIF)

Figure S4 Functions of ecoptic constitutively active and endogenous Eomes in notochord development. (A) Injection of 25 pg *eomes-VP16* frequently causes rescue of notochord formation in MZ*mid* mutants. (B) Injection of *eomes-enR* does not inhibit notochord formation in wild-type embryos. Arrowheads indicate notochords in injected embryos.
(TIF)

Table S1 Overexpression of *mid* causes mild defects compared to *sur* and *FoxH1*. Embryos were scored at 24 hpf; results are expressed as percentages of the corresponding totals. Mild defects include: small eyes, eyes of unequal sizes, narrow head, kinked notochord, wavy notochord, ventral body curvature. Severe defects include: one unilateral eye, cyclopia, no eyes, loss of head structures, ventrally displaced notochord with irregular morphology, no notochord, reduced trunk or tail structures. Catastrophic defects include dead embryos, unrecognizable tissue masses, embryos too poorly developed to accurately describe. Note that injection of *mid* mRNA produces much less severe effects in wild-type embryos at either dose compared to *FoxH1* or *sur* mRNA. By chi-square analysis, the result of injecting 50 pg of *FoxH1* or *sur* mRNA is not significantly different from the other (p-value = 0.20012). However, *mid* mRNA injection is statistically significantly different from *FoxH1* mRNA (p-value = 4.6E-35) or *sur* mRNA injections (p-value = 1.34E-33).
(DOC)
