# Supplementary figures and images for: Nodal-Dependent Mesendoderm Specification Requires the Combinatorial Activities of FoxH1 and Eomesodermin
Source: PLoS Genet. 2011 May 26;7(5):e1002072. doi: 10.1371/journal.pgen.1002072 (PMC3102743; doi:10.1371/journal.pgen.1002072)

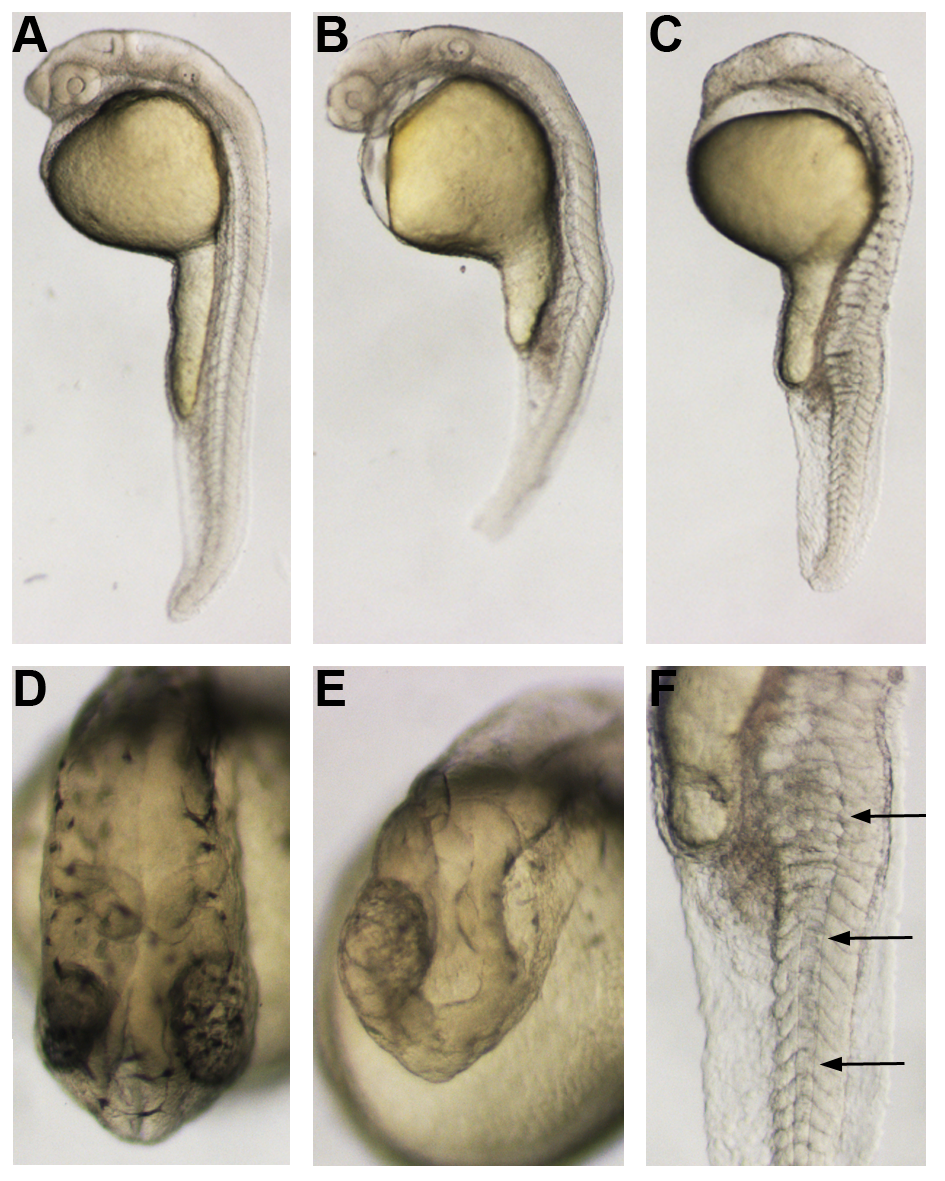

Supplement: Figure S1 — Defects caused by overexpression of FoxH1, sur, and mid mRNA. Representative images of common phenotypes observed upon FoxH1, sur, and mid overexpression in wild-type embryos. (A) Embryo injected with 100 pg mid mRNA exhibiting a wild-type appearance at 24 hpf. (B) Embryo injected with 100 pg mid mRNA exhibiting a wavy notochord. (C) Embryo injected with 50 pg FoxH1 mRNA exhibiting a loss of eyes and head structures, and a morphologically irregular notochord (also see panel F). (D) Dorsal anterior view of an embryo injected with 50 pg sur mRNA exhibiting eyes of unequal sizes. (E) Dorsal anterior view of an embryo injected with 50 pg FoxH1 mRNA exhibiting a single unilateral eye. (F) Enlarged portion of embryo in panel C at the level of the yolk extension. Arrows indicate the abnormal notochord. (TIF) [file pgen.1002072.s001.tif]

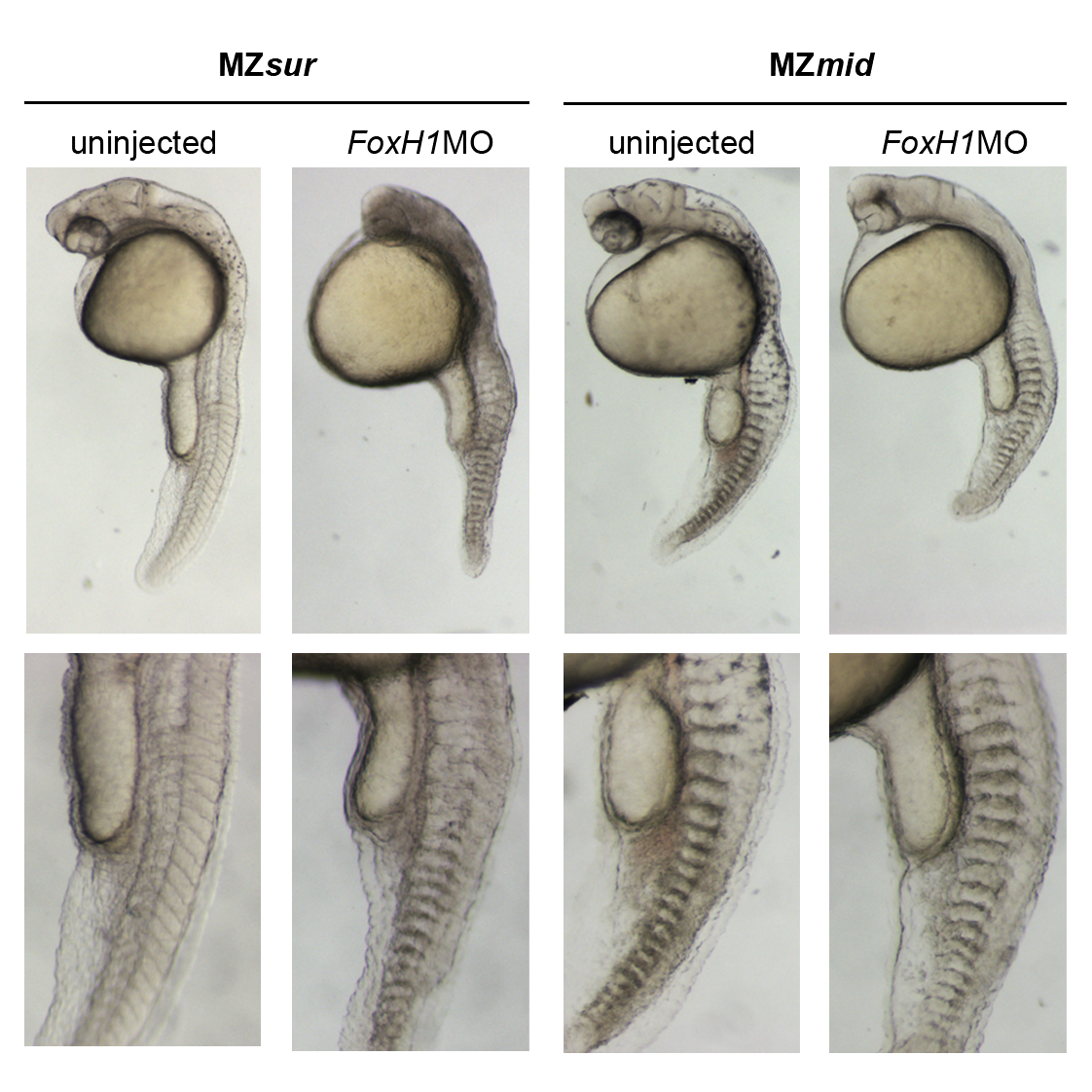

Supplement: Figure S2 — Effects of FoxH1 knockdown on MZsur and MZmid mutants. MZsur and MZmid embryos were injected with 4 ng FoxH1MO to assess the effect of inhibiting production of mutant FoxH1 proteins on development. A majority of injected MZsur embryos lack notochords (115/187 without notochords); injection into MZmid mutants never rescues notochord formation (0/182 with notochords). 15/182 injected MZmid mutants had slight defects including delayed development and nonspecific necrosis which may be injection artifacts. 4/182 displayed midline bifurcations. In 5/182 embryos, injection of FoxH1MO into MZmid caused splitting of the normally fused eye field into two eyes. This effect is most likely related to the published role of the Foxh1 DNA-binding domain in inhibiting the mixl1 promoter via recruitment of Gsc protein in mouse _ [41] (see Discussion for more details). (TIF) [file pgen.1002072.s002.tif]

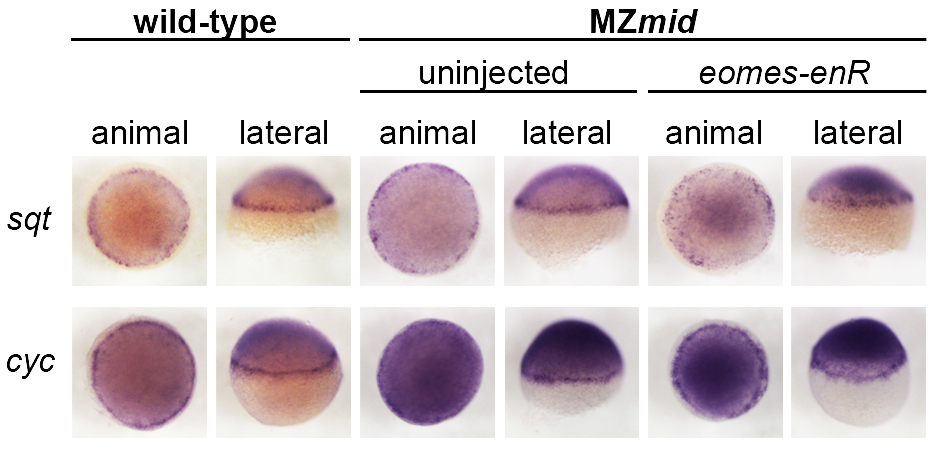

Supplement: Figure S3 — Nodal ligand expression in MZmid mutants Pre-gastrulation expression of cyc and sqt were analyzed in uninjected and eomes-enR-injected MZmid embryos. Expression resembles that in wild-type embryos, though at lower levels, and is unaffected by Eomes inhibition. (TIF) [file pgen.1002072.s003.tif]

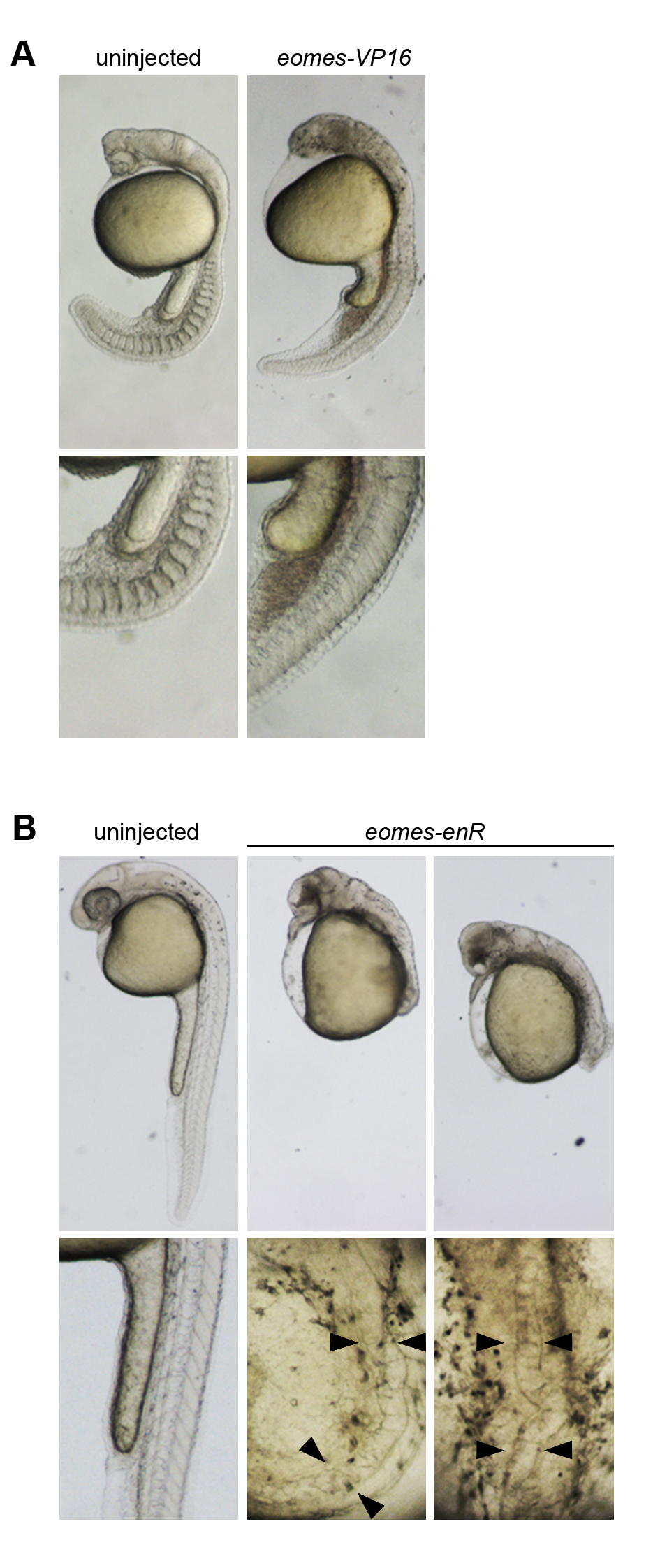

Supplement: Figure S4 — Functions of ecoptic constitutively active and endogenous Eomes in notochord development. (A) Injection of 25 pg eomes-VP16 frequently causes rescue of notochord formation in MZmid mutants. (B) Injection of eomes-enR does not inhibit notochord formation in wild-type embryos. Arrowheads indicate notochords in injected embryos. (TIF) [file pgen.1002072.s004.tif]
