# Supplementary material for: Nodal-Dependent Mesendoderm Specification Requires the Combinatorial Activities of FoxH1 and Eomesodermin
Source: PLoS Genet. 2011 May 26;7(5):e1002072. doi: 10.1371/journal.pgen.1002072 (PMC3102743; doi:10.1371/journal.pgen.1002072)
Supplement: Table S1 — Overexpression of mid causes mild defects compared to sur and FoxH1. Embryos were scored at 24 hpf; results are expressed as percentages of the corresponding totals. Mild defects include: small eyes, eyes of unequal sizes, narrow head, kinked notochord, wavy notochord, ventral body curvature. Severe defects include: one unilateral eye, cyclopia, no eyes, loss of head structures, ventrally displaced notochord with irregular morphology, no notochord, reduced trunk or tail structures. Catastrophic defects include dead embryos, unrecognizable tissue masses, embryos too poorly developed to accurately describe. Note that injection of mid mRNA produces much less severe effects in wild-type embryos at either dose compared to FoxH1 or sur mRNA. By chi-square analysis, the result of injecting 50 pg of FoxH1 or sur mRNA is not significantly different from the other (p-value = 0.20012). However, mid mRNA injection is statistically significantly different from FoxH1 mRNA (p-value = 4.6E-35) or sur mRNA injections (p-value = 1.34E-33). (DOC) [file pgen.1002072.s005.doc]

**Table S1. Overexpression of *mid* causes mild defects compared to *sur* and *FoxH1***

|  |  | **50pg** | | | **100pg** | | |
| --- | --- | --- | --- | --- | --- | --- | --- |
| **defects observed** | **uninjected** | ***FoxH1*** | ***sur*** | ***mid*** | ***FoxH1*** | ***sur*** | ***mid*** |
| wild-type | 97.5 | 25.6 | 17.6 | 85.6 | 4.9 | 2.7 | 28.0 |
| mild | 0 | 35.0 | 26.4 | 13.2 | 6.6 | 10.7 | 26.0 |
| severe | 0.8 | 34.1 | 48.5 | 0.6 | 37.6 | 46.7 | 15.5 |
| catastrophic | 1.7 | 6.1 | 6.6 | 0.6 | 50.8 | 40.0 | 30.6 |
|  | n=119 | n=297 | n=136 | n=167 | n=61 | n=75 | n=193 |

Embryos were scored at 24 hpf; results are expressed as percentages of the corresponding totals. Mild defects include: small eyes, eyes of unequal sizes, narrow head, kinked notochord, wavy notochord, ventral body curvature. Severe defects include: one unilateral eye, cyclopia, no eyes, loss of head structures, ventrally displaced notochord with irregular morphology, no notochord, reduced trunk or tail structures. Catastrophic defects include dead embryos, unrecognizable tissue masses, embryos too poorly developed to accurately describe. Note that injection of *mid* mRNA produces much less severe effects in wildtype embryos at either dose compared to *FoxH1* or *sur* mRNA. By chi-square analysis, the result of injecting 50pg of *FoxH1* or *sur* mRNA is not significantly different from the other (p_value 0.20012). However, *mid* mRNA injection is statistically significantly different from *FoxH1*mRNA (p_value 4.6E-35) or *sur* mRNA injections (p_value 1.34E-33).
